# The interaction discrepancy model: a theoretical framework for understanding person-environment interactions

**DOI:** 10.3389/fpsyg.2025.1554567

**Published:** 2025-10-27

**Authors:** Robin Umbra, Ulrike Fasbender

**Affiliations:** Chair of Business and Organizational Psychology, University of Hohenheim, Stuttgart, Germany

**Keywords:** interaction discrepancy model, person-environment interactions, conceptual framework, theoretical model development, morality, moral appraisal, moral emotions

## Abstract

This manuscript introduces the Interaction Discrepancy Model (IDM), a theoretical framework designed to enhance our understanding of person-environment interactions. Traditional models often overlook the dynamic, iterative, and feedback-driven nature of these interactions, typically focusing on episodic and isolated psychological processes and conscious mechanisms. The IDM addresses these limitations by integrating the dynamics of cognitive, affective, and behavioral processes at both conscious and non-conscious levels. The model outlines an eight-stage process: (1) perception, (2) interaction construal, (3) verification, (4) congruence/discrepancy, (5) appraisal, (6) autoregulatory response, (7) action plan, and (8) feedback. This comprehensive approach seeks to explain the varied responses observed in empirical research and real-life scenarios. The IDM’s applicability extends across multiple contexts, including aggression, delinquency, conflict management, and industrial-organizational psychology, emphasizing the critical role of perceived discrepancies in triggering affective and behavioral responses. By incorporating contextual factors and providing a structured framework for falsifiability, the IDM offers a robust tool for future research and practical applications. This model significantly advances the theoretical literature on person-environment interactions, providing a holistic understanding that captures the complexity of human experience.

## Introduction

Person-environment interactions, encompassing exchanges between persons and other people, animals, inanimate objects, or abstract concepts (American Psychological Association, 2024), are fundamental to human experience. Despite their importance (Blau, 1964; Higgins, 1987; Lazarus, 1991), these interactions’ dynamic nature remains poorly understood (Campos et al., 2011; Cottingham, 2024; Fischer and van Kleef, 2010). This manuscript presents a novel conceptual framework aimed at incrementally enhancing our understanding of the dynamic interactions between individuals and their environments. Grasping these interactional dynamics is essential for predicting the outcomes of such interactions and identifying ways to influence them for the betterment of humanity (Dillon et al., 2011; Halperin, 2014; Maroney and Gross, 2014).

Numerous theoretical models have explored these interactions (Blau, 1964; Frese and Zapf, 1994; Hacker, 1985; Lazarus, 1991; Roseman, 1984; Scherer, 2009; Vroom, 1964; Weiss and Cropanzano, 1996), yet they often come with limitations to capture their full dynamics (see Karstedt, 2016; Kuppens, 2015; Schoebi and Randall, 2015). Typically, these models treat interactions as episodic and static (e.g., Vroom, 1964; Weiss and Cropanzano, 1996), overlooking their continuous, iterative, and feedback-driven nature (Hollenstein, 2015). Additionally, they usually focus on one or two psychological processes (cognition, affect, behavior) at a time, neglecting the complexity of interactions involving all three processes (e.g., Blau, 1964; Vroom, 1964; Weiss and Cropanzano, 1996). When all three are considered, it is often to a limited degree, leaving scholars to question how such explanations can account for the diversity in cognitive, affective, and behavioral responses observed in empirical research (Pessoa, 2010; Scherer, 2013; Umbra & Fasbender, in press). Furthermore, current models typically focus on conscious processes (e.g., Blau, 1964; Higgins, 1987; Vroom, 1964), disregarding recent empirical developments that highlight the role of non-conscious processes (Frijda, 2009; Mallon and Nichols, 2011; Winkielman, 2010). Thus, while existing frameworks have advanced our understanding of person-environment interactions, their fragmented approach underscores the need for a holistic model to address these limitations.

To address these aspects, we propose the Interaction Discrepancy Model (IDM). This model integrates prior theoretical and empirical research to create a more holistic understanding of person-environment interactions, accounting for their dynamic nature. The IDM outlines an eight-stage process: (1) perception of person-environment interaction, (2) interaction construal, (3) verification process, (4) congruence/discrepancy, (5) appraisal process, (6) autoregulatory response, (7) action plan, and (8) feedback. This process aims to explain the dynamic, iterative, and feedback-driven nature of these interactions, incorporating the interplay of cognitive, affective, and behavioral processes at both conscious and non-conscious levels.

The IDM seeks to answer different key questions: How do person-environment interactions unfold and evolve (Boiger and Mesquita, 2012)? Why and how do these interactions lead to (or fail to do so) a wide range of cognitive, affective, and behavioral responses and adaptations (Horberg et al., 2011; Izard, 2011; Packard and Schultz, 2023)? How do cognitive, affective, and behavioral processes within a person-environment interaction influence each other (Castelfranchi and Miceli, 2009; Harré, 2009; Helm, 2009)? By systematically addressing these questions, the IDM aims to provide a robust conceptual framework that deepens our understanding of the dynamics inherent in person-environment interactions and lays the groundwork for future scientific and practical applications (Picard, 2010).

Our work aims to make three significant contributions to the theoretical literature on person-environment interactions. First, we integrate and advance propositions from traditional theoretical models (Blau, 1964; Frese and Zapf, 1994; Hacker, 1985; Lazarus, 1991; Roseman, 1984; Scherer, 2009; Vroom, 1964; Weiss and Cropanzano, 1996) to account for the dynamic, iterative, and feedback-driven nature of person-environment interactions, addressing the question of how these interactions unfold and evolve (Boiger and Mesquita, 2012). We propose an eight-stage process model that starts with the perception of person-environment interaction, followed by interaction construal, verification process, detection of discrepancies, appraisal process, autoregulatory responses, action plan, and ends with a feedback loop that influences the initial stages until certain conditions are met. This model aims to provide researchers with a more holistic understanding of the dynamic, iterative, and feedback-driven nature of person-environment interactions.

Second, we elucidate the complex interplay of cognitive, affective, and behavioral processes in person-environment interactions to explain why and how these interactions (fail to) lead to a wide range of cognitive, affective, and behavioral responses and adaptations (Horberg et al., 2011; Izard, 2011; Packard and Schultz, 2023). We propose that these responses and adaptations occur to reconcile current interactions (Is-State) with expected interactions (Ought-State), aligning or improving current interactions with expected ones. This reconciliation occurs through a dynamic, iterative, and feedback-driven process involving the perception, appraisal, and response to discrepancies. The central proposition of the IDM is that the perception of discrepancies in person-environment interactions prompts persons to deploy cognitive, affective, and behavioral processes to address these discrepancies.

Third, we integrate recent empirical developments in neuroscience and cognitive psychology (e.g., Gazzaley and Nobre, 2012; Kozhevnikov et al., 2007; Nie et al., 2017) to elucidate the complex interplay of conscious and non-conscious mechanisms within person-environment interactions (Frijda, 2009; Mallon and Nichols, 2011; Winkielman, 2010). We propose that these processes are influenced by both overt and subliminal sensory perceptions and feedback responses, which direct changes in cognitive, affective, and behavioral processes. This aims to provide researchers with a more holistic understanding of how cognitive, affective, and behavioral processes within person-environment interactions influence each other.

## Literature review

### Review of existing models

Person-environment interactions have been extensively explored through various traditional models, including models of cognitive appraisal (Lazarus, 1991; Roseman, 1984; Scherer, 2009), self-discrepancy (Higgins, 1987), social exchange (Blau, 1964), expectancy-value (Vroom, 1964), affective events (Weiss and Cropanzano, 1996), and action regulation (Hacker, 1985; Frese and Zapf, 1994). These models have advanced our understanding of these interactions but also have limitations that restrict the inferences drawn from them.

#### Cognitive appraisal models

Cognitive appraisal models, such as those proposed by Lazarus (1991), Roseman (1984), and Scherer (2009), assert that person-environment interactions involve an iterative interplay of cognition, affect, and behavior. These models posit that the cognitive assessment of a person-environment interaction leads to an emotional response, which in turn guides behavior aimed at coping with the favorable or unfavorable states resulting from the interaction. However, these models often treat psychological processes beyond cognition (and sometimes affect) to a limited degree, constraining their ability to account for the diversity in affective, and behavioral responses observed in empirical research (Umbra and Fasbender, 2025). Therefore, cognitive appraisal models require theoretical extension to address the wide range of behavioral responses and adaptations documented in empirical research.

#### Self-discrepancy models

Self-discrepancy models, such as those proposed by Higgins (1987), highlight the iterative and feedback-driven nature of person-environment interactions, focusing on perceived discrepancies between a person’s actual, ideal, and ought selves. However, these models fall short in addressing the dynamic nature of person-environment interactions, given that discrepancies between a person’s actual, ideal, and ought selves are long-term processes that are unlikely to change quickly. Additionally, these models often constrain their treatment of adaptational processes to the conscious level, disregarding recent empirical developments highlighting the role of non-conscious processes. Therefore, self-discrepancy models require theoretical extension to address the dynamic nature of person-environment interactions and the role of non-conscious processes.

#### Social exchange models

Social exchange models, such as those proposed by Blau (1964), emphasize the dynamic and iterative interplay of cognition and behavior to maximize the benefit derived from person-environment interactions. However, these models often neglect the role of affective responses and adaptations observed in empirical studies and constrain their treatment of interaction processes to the conscious level. Therefore, social exchange models require theoretical extension to address the wide range of affective responses and adaptations observed in empirical studies and the role of non-conscious processes.

#### Expectancy-value models

Expectancy-value models, such as those proposed by Vroom (1964), assert that person-environment interactions involve a dynamic interplay of cognition, affect (specifically its motivational component), and behavior. These models posit that the cognitive assessment of an expected person-environment interaction determines the motivational states and subsequent behaviors. However, these models often conceptualize person-environment interactions as episodic and static, addressing the wide range of affective behavioral responses and adaptations only to a limited degree. Additionally, these models typically constrain their treatment of interaction processes to the conscious level. Therefore, expectancy-value models require theoretical extension to address the iterative and feedback-driven nature of person-environment interactions and the role of non-conscious processes.

#### Affective events models

Affective events models, such as those proposed by Weiss and Cropanzano (1996), assert that person-environment interactions involve a dynamic interplay of cognition, affect, and behavior. These models posit that the cognitive assessment of a person-environment interaction leads to an emotional response, which guides behavior aimed at coping with the interaction’s outcomes. However, these models often treat person-environment interactions as episodic and static, neglecting their iterative and feedback-driven nature. Additionally, these models typically constrain their treatment of interaction processes to the conscious level. Therefore, affective events models require theoretical extension to address the iterative and feedback-driven nature of person-environment interactions and the role of non-conscious processes.

#### Action regulation models

Action regulation models, such as those proposed by Hacker (1985) and expanded by Frese and Zapf (1994), assert that person-environment interactions involve a dynamic, iterative, and feedback-driven interplay of cognition, affect, and behavior. However, these models often constrain themselves to task-related processes and neglect the wide range of affective responses and adaptations observed in empirical studies, particularly positive emotions. Additionally, these models typically constrain their treatment of interaction processes to the conscious level. Therefore, action regulation models require theoretical extension to address the broad scope of potential contexts of person-environment interactions and the role of non-conscious processes.

### Summary

While traditional models have significantly advanced our understanding of person-environment interactions, each one has its limitations. Cognitive appraisal models have limitations in addressing the full spectrum of behavioral responses and adaptations observed in empirical research (Lazarus, 1991; Roseman, 1984; Scherer, 2009). Self-discrepancy models overlook the dynamic nature of these interactions and the influence of non-conscious processes (Higgins, 1987). Social exchange models have some issues covering the range of affective responses and adaptations found in studies, nor the role of non-conscious processes (Blau, 1964). Expectancy-value models seem to miss the iterative and feedback-driven aspects of person-environment interactions, as well as the role of non-conscious processes (Vroom, 1964). Similarly, affective events models do not sufficiently address these iterative and feedback-driven elements (Weiss and Cropanzano, 1996). Finally, action regulation models seem to lack the scope to encompass the varied contexts of person-environment interactions and the influence of non-conscious processes (Hacker, 1985; Frese and Zapf, 1994). These shortcomings necessitate a novel conceptual model—such as the Interaction Discrepancy Model (IDM)—that comprehensively captures the complexity of person–environment interactions and addresses the limitations of prior models.

### Theoretical integration and extension

The Interaction Discrepancy Model (IDM) builds on these foundational theoretical models by integrating their strengths and addressing their limitations. By synthesizing these established theoretical concepts, the IDM provides a more holistic and dynamic perspective on person-environment interactions.

The IDM incorporates appraisal processes from cognitive appraisal models as a core component (e.g., Lazarus, 1991; Roseman, 1984; Scherer, 2009). It adopts the rationale behind the relationship between these appraisal processes and affective and behavioral responses, while extending these models to better account for the diverse responses observed in empirical research.

The IDM also integrates the discrepancy detection and resolution processes from self-discrepancy models (e.g., Higgins, 1987). It expands on how these processes are resolved and addresses the dynamic nature of person-environment interactions, extending the treatment of adaptation processes to the unconscious level.

Additionally, the IDM incorporates benefit-directed processes from social exchange models (e.g., Blau, 1964). It adopts the rationale behind achieving these benefits and extends the models to include the role of affective responses and adaptations at the non-conscious level.

From expectancy-value models, the IDM integrates the rationale that assessments of expected person-environment interactions determine motivational states and subsequent behaviors (e.g., Vroom, 1964). It extends these models to address the wide range of affective and behavioral responses and adaptations on both conscious and non-conscious levels.

The IDM also draws from affective events models concerning the relationships between person-environment interactions, affective responses, and behavioral responses (e.g., Weiss and Cropanzano, 1996). It extends these models to address the iterative, feedback-driven nature of person-environment interactions and the role of non-conscious processes.

Finally, the IDM incorporates dynamic, iterative, and feedback-driven processes from action regulation models (e.g., Hacker, 1985; Frese and Zapf, 1994). It extends these models to address the broad scope of potential contexts for person-environment interactions and the role of non-conscious processes.

### Approach and model construction

The Interaction Discrepancy Model (IDM) was developed through a structured conceptual synthesis that combined theory review, meta-empirical integration, and dialectical model refinement. The process began with a scoping review of existing models that address person–environment interactions, focusing on conceptual frameworks in which an individual interacts with another entity (e.g., environments, systems, other people). These models, discussed in the preceding section, served as a foundational set of incumbent approaches.

Concurrently, three complementary literature streams were systematically analyzed to inform and test the model structure. First, a comprehensive narrative review was conducted of all peer-reviewed review articles on moral emotions—specifically anger, disgust, shame, and guilt—retrieved via Scopus from 1978 to 2025, yielding 378 articles in English, German, and Russian. Second, 281 review and conceptual papers on the psychotherapeutic regulation and transformation of these same emotions were selected from leading clinical and counseling journals, including Journal of Behavioral and Cognitive Therapy, Clinical Social Work Journal, and Behavior Research and Therapy. This stream supported the goal of generalizing the model to applied contexts, such as forensic or psychiatric populations. Third, a meta-empirical synthesis of 72 meta-analyses published in Emotion Review, Cognition & Emotion, and Motivation & Emotion was conducted to identify common antecedents, regulatory mechanisms, and outcome patterns across cognitive, affective, and behavioral processes.

Following this review phase, the model was constructed through an iterative synthesis process. Incumbent models were compared against insights from the empirical and meta-analytical literature to assess theoretical alignment. Where discrepancies arose—whether due to conflicting findings, insufficient explanatory scope, or internal logical inconsistency—these were addressed using a dialectical approach inspired by Socratic reasoning (Kreeft, 2014). Contradictions were resolved through stepwise reformulation, and each emerging model component was re-examined in light of the full evidence base. This cyclical process of falsifiability testing and logical reconstruction continued until all stages of the IDM demonstrated coherence with both prior theory and observed patterns in the literature. The resulting model architecture and stage sequence thus reflect an inductive conceptual derivation grounded in theoretical pluralism and meta-analytic integration.

## Theoretical framework

**Figure 1:**
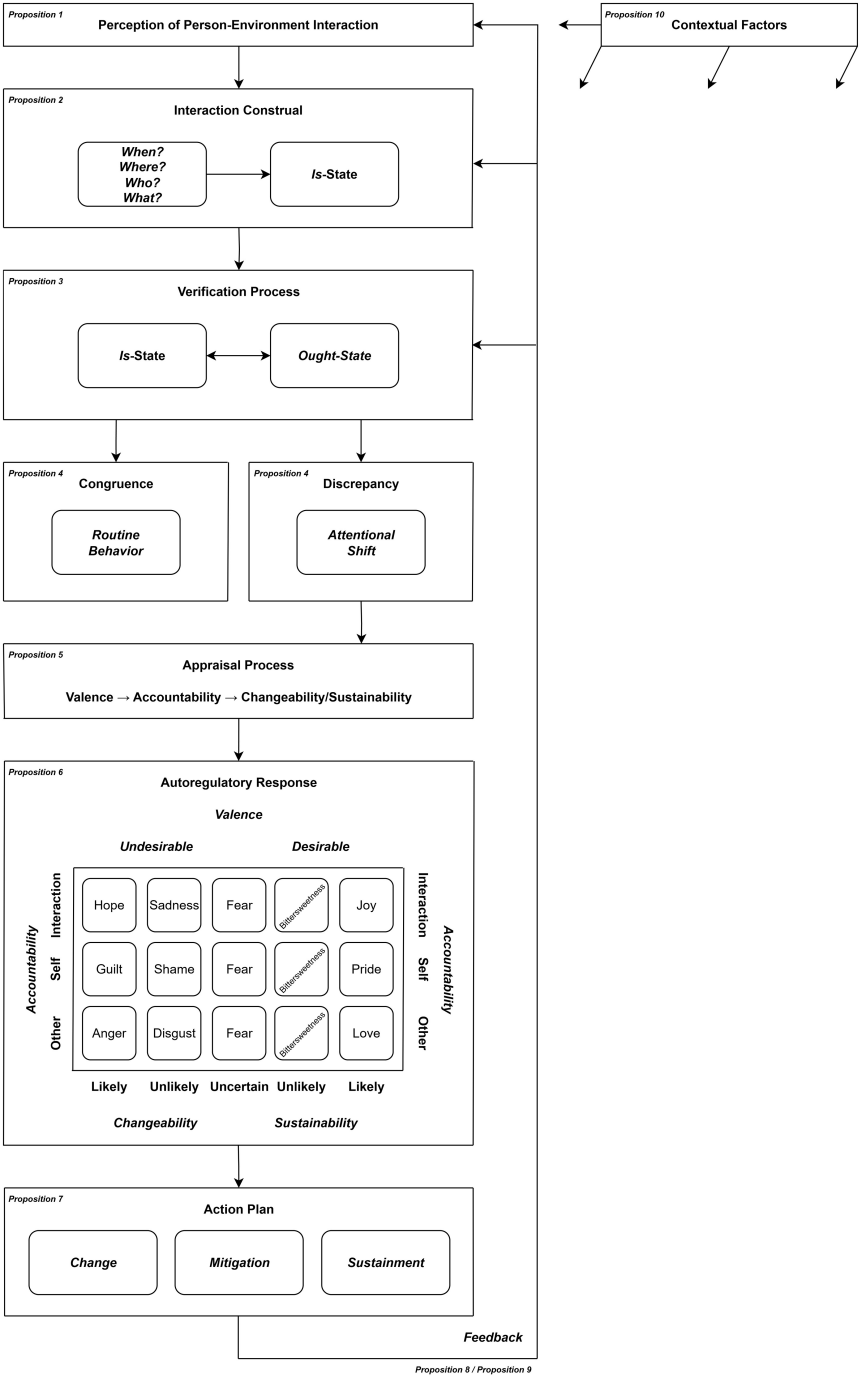
Interaction discrepancy model.

The Interaction Discrepancy Model (IDM) provides a detailed framework for understanding and managing person-environment interactions. The model identifies eight critical stages in the interaction process: perception of person-environment interaction, interaction construal, verification process, congruence/discrepancy, appraisal process, autoregulatory response, action plan, and feedback. Contextual factors also influence these stages. This section explores each stage in detail to enhance our understanding of these processes (see Figure 1).

### Stage 1: perception of person-environment interaction

The perception of person-environment interactions, whether occurring individually or concurrently, forms the foundation of the IDM and encompasses both conscious and non-conscious processes. Conscious interactions involve active engagement with one’s surroundings, whether initiated by the person or the environment. For example, a person might deliberately focus on specific environmental aspects, altering their sensory experience or mental state. Non-conscious interactions, conversely, occur without the person’s awareness, such as the nervous system’s automatic adjustments to maintain balance or detect environmental changes. These interactions set the stage for noticing and addressing discrepancies, defining the dynamic and iterative nature of person-environment interactions. Therefore, our proposition is that the perception of person-environment interactions, involving both conscious and non-conscious processes and changes initiated by either the individual or the environment, forms the foundation of interactions between individuals and their environments (*Proposition 1*). Prior research on sensory perception (Engel et al., 2001; Proulx et al., 2014; Stein and Stanford, 2008) and environmental psychology (Saarimäki, 2021; Schreuder et al., 2016; Spence, 2020) supports this proposition.

### Stage 2: interaction construal

Interaction construal involves forming a comprehensive mental picture of the interaction based on data collected from initial person-environment interactions. This stage focuses on organizing data into a coherent framework without interpretation. For instance, observing a snow fox in a snowy landscape raises questions about timing, location, participants, and plot, helping to build a clear mental representation of the interaction. Therefore, our proposition is that following the perception of person-environment interactions, persons form a comprehensive mental picture of these interactions by organizing data into a coherent framework without interpretation (*Proposition 2*). Research on cognitive frameworks supports this proposition (Gazzaley and Nobre, 2012; Kozhevnikov et al., 2007; Nie et al., 2017).

### Stage 3: verification process

The verification process involves comparing the current interaction (Is-State) with the expected interaction (Ought-State) to identify discrepancies within the current person-environment interaction (see also Higgins, 1987). This comparison considers context-specific factors such as timing, location, participants, and plot. By evaluating these elements without judgment, persons can determine whether the current interaction aligns with expectations or deviates from them, setting the stage for further cognitive and emotional processing. Therefore, our proposition is that after forming a comprehensive mental picture of interactions, individuals compare these current interactions (Is-State) with expected interactions (Ought-State) to identify discrepancies, considering context-specific factors such as timing, location, participants, and plot, all without interpretation (*Proposition 3*). This proposition is supported by research on cognitive processes (Evans and Stanovich, 2013; Gigerenzer and Gaissmaier, 2011; Sunstein, 2005).

### Stage 4: congruence and discrepancy

This stage examines whether the current interaction matches the expected interaction. Congruence occurs when there is alignment, allowing routine behavior to continue without additional cognitive effort. Discrepancy, however, prompts persons to address the mismatch, focusing their attention on addressing the discrepancy. Understanding the alignment or misalignment between the Is-State and Ought-State is critical for subsequent responses. Notably, this stage still proceeds without interpretation. Therefore, our proposition is that after comparing current interactions (Is-State) with expected interactions (Ought-State), a person either continues routine behavior without additional cognitive effort if there is congruence or focuses on the misaligned person-environment interaction if there is a discrepancy, all without interpretation (*Proposition 4*). Research on cognitive dissonance (Festinger, 1957) and related concepts supports this proposition (Elliot and Devine, 1994; Harmon-Jones, 2000; van Veen et al., 2009).

### Stage 5: appraisal process

The appraisal process evaluates identified discrepancies through a structured four-step method: assessing the valence of the discrepancy based on its moral desirability (Jensen, 2015; Shweder et al., 1997), determining responsibility, evaluating changeability or sustainability, and synthesizing these evaluations into an overall appraisal outcome. For example, a desirable discrepancy might be assessed for its potential to be sustained, while an undesirable one is evaluated for its likelihood of change. These appraisals guide the person’s subsequent responses. Therefore, our proposition is that after shifting attention toward a discrepancy within a person-environment interaction, individuals evaluate the identified discrepancies using a structured four-step method: assessing the valence based on moral desirability, determining responsibility, evaluating changeability or sustainability, and synthesizing these evaluations into an overall appraisal outcome (*Proposition 5*). Research on appraisal processes supports this proposition (Ellsworth, 2013; Scherer, 2009; Siemer et al., 2007).

### Stage 6: autoregulatory response

Following the appraisal outcome, both modulatory and motivational changes manifest (Frijda, 1987), culminating in observable autoregulatory responses. These responses, in their latent form, are identified as emotions. For example, appraising an undesirable yet changeable discrepancy attributed to the environment may lead to an increased heart rate (modulatory component) and an urge for retribution (motivational component; Lazarus, 1991), collectively forming the emotion of anger. The degree to which the components of the appraisal outcome are expressed determines the variation in autoregulatory responses, potentially resulting in emotional blends. The larger the discrepancy, the stronger is the autoregulatory response and the corresponding emotion. Therefore, our proposition is that following the appraisal outcome, modulatory and motivational changes occur, regulated by the expressions and combinations of the appraisal components, forming the latent construct we identify as an emotion (*Proposition 6*). This proposition is corroborated by research on intrapersonal regulation (Kreibig, 2010; Lang and Bradley, 2010; Roseman, 2013).

#### Response categorization

Table 1 provides a detailed breakdown of the relationship between the components of the appraisal outcome and the ensuing autoregulatory responses. The categorization within the table is based on previous research on appraisal theory (Roseman, 1984; Frijda, 1987; Lazarus, 1991).

**Table 1:** Input and output factors of the appraisal process.

Input factors				Output factors		
Valence	Accountability	Changeability	Sustainability	Modulation	Motivation	Latent construct
Undesirable	Other	Likely		Excitation	Retributive	Anger
Undesirable	Self	Likely		Excitation	Penitent	Guilt
Undesirable	Interaction	Likely		Excitation	Prospective	Hope
Undesirable	Other	Unlikely		Inhibition	Terminative	Disgust
Undesirable	Self	Unlikely		Inhibition	Isolative	Shame
Undesirable	Interaction	Unlikely		Inhibition	Conservative	Sadness
Undesirable	Other	Uncertain		Excitation	Preparative	Fear
Undesirable	Self	Uncertain		Excitation	Preparative	Fear
Undesirable	Interaction	Uncertain		Excitation	Preparative	Fear
Desirable	Other		Uncertain	Excitation	Preparative	Fear
Desirable	Self		Uncertain	Excitation	Preparative	Fear
Desirable	Interaction		Uncertain	Excitation	Preparative	Fear
Desirable	Other		Likely	Excitation	Affiliative	Love
Desirable	Self		Likely	Excitation	Exhibitive	Pride
Desirable	Interaction		Likely	Excitation	Maintaining	Joy
Desirable	Other		Unlikely	Inhibition	Reflective	Bittersweetness
Desirable	Self		Unlikely	Inhibition	Reflective	Bittersweetness
Desirable	Interaction		Unlikely	Inhibition	Reflective	Bittersweetness

Valence represents the internal moral value associated with a discrepancy (Shweder et al., 1997). It is categorized as either desirable, indicating a positive moral value, or undesirable, indicating a negative moral value.

Accountability refers to the source of responsibility for a discrepancy and is divided into three categories (Roseman, 1984): self, where the individual takes personal responsibility; other, where responsibility is attributed to other people, animals, inanimate objects, or abstract concepts; and interaction, where responsibility is attributed to the interaction between the individual and the environment or others.

Changeability pertains only to undesirable discrepancies and indicates the perceived likelihood that the situation can be changed. It is classified as likely, implying a high probability of change; unlikely, implying a low probability of change; and uncertain, indicating an ambiguous or unknown likelihood of change.

Sustainability pertains only to desirable discrepancies and refers to the ability to maintain or sustain a change over time. It is categorized as likely, implying a high probability of sustaining; unlikely, implying a low probability of sustaining; and uncertain, indicating an ambiguous or unknown likelihood of sustaining.

#### Undesirable discrepancies with likely changeability

When an undesirable discrepancy with likely changeability is attributed to another person, modulatory and motivational changes activate the autonomic nervous system and prompt a retributive response. This combined output forms the observed construct, while the latent construct is termed “Anger.” Similarly, if the discrepancy is attributed to oneself, these changes activate the autonomic nervous system and elicit a penitent response, encapsulated by the latent construct “Guilt.” For discrepancies attributed to the interaction, these changes again activate the autonomic nervous system and induce a prospective response, represented by the latent construct “Hope.”

#### Undesirable discrepancies with unlikely changeability

When an undesirable discrepancy with unlikely changeability is attributed to another person, modulatory and motivational changes inhibit the autonomic nervous system and provoke a terminative response, encapsulated by the latent construct “Disgust.” If the discrepancy is attributed to oneself, these changes inhibit the autonomic nervous system and lead to an isolative response, captured by the latent construct “Shame.” For discrepancies attributed to the interaction, the same changes inhibit the autonomic nervous system and result in a conservative response, represented by the latent construct “Sadness.”

#### Discrepancies with uncertain outcomes

When encountering discrepancies with uncertain outcomes, whether characterized by undesirable changeability or desirable sustainability, the response pattern remains consistent across various attributions of accountability. In each scenario—whether the discrepancy is attributed to another person, oneself, or the interaction—modulatory and motivational changes trigger the autonomic nervous system and induce a preparative stance, encapsulated by the latent construct “Fear.” Therefore, regardless of the nature of the discrepancy or its attribution, the preparative response characterized by “Fear” remains uniformly consistent.

#### Desirable discrepancies with likely sustainability

When a desirable discrepancy with likely sustainability is attributed to another person, modulatory and motivational changes activate the autonomic nervous system and prompt an affiliative response, encapsulated by the latent construct “Love.” If the discrepancy is attributed to oneself, these changes activate the autonomic nervous system and lead to an exhibiting response, represented by the latent construct “Pride.” For discrepancies attributed to the interaction, the same changes activate the autonomic nervous system and result in a maintaining response, captured by the latent construct “Joy.”

#### Desirable discrepancies with unlikely sustainability

When a desirable discrepancy with unlikely sustainability is attributed to another person, oneself, or the interaction, modulatory and motivational changes inhibit the autonomic nervous system and prompt a reflective response. This integrated response is consistently encapsulated by the latent construct “Bittersweetness.”

### Stage 7: action plan

The Action Plan stage involves developing and implementing a strategy to address the identified discrepancy. This strategy is guided by the psychological component of the autoregulatory response, which provides direction, and supported by the physiological component of the autoregulatory response, which provides physical adaptation. The action plan can include change-oriented responses to reduce the discrepancy, mitigation-oriented responses to lessen its impact, or sustainment-oriented responses to maintain beneficial discrepancies. Successful implementation of the action plan is crucial for managing the discrepancy effectively. Therefore, our proposition is that following the autoregulatory response, individuals develop and implement strategies to address identified discrepancies, guided by the motivational changes and supported by the modulatory changes, using change-oriented, mitigation-oriented, or sustainment-oriented actions (*Proposition 7*). Research on interpersonal regulation supports this proposition (Folkman et al., 1986; Fredrickson, 2001; Gross, 2015).

### Stage 8: feedback

The Feedback stage captures the outcomes of the action plan, providing insights into its effectiveness in addressing the discrepancy. By observing the effects on person-environment interactions and evaluating changes in the perceived interaction, persons can determine whether the discrepancy has been managed as expected. This feedback informs the management of current and future interactions, creating a dynamic and iterative process aimed at reconciling the Is-State with the Ought-State. Therefore, our proposition is that following the enactment of the action plan, individuals evaluate changes in person-environment interactions to determine if the discrepancies have been managed as expected (*Proposition 8*). Research on feedback mechanisms supports this proposition (Barsade, 2002; Cialdini and Goldstein, 2004; Van Kleef et al., 2004).

The feedback can correlate with the experience of positive or negative affect, depending on whether the discrepancy has been managed as expected. Successful management of the discrepancy may result in positive affect, such as satisfaction or relief. Conversely, if the discrepancy persists or worsens, negative affect, such as frustration or disappointment, may occur. This affective response further influences the management of current and future interactions. Therefore, our proposition is that during the evaluation of the action plan’s effectiveness, individuals experience positive or negative affect based on their interpretation of whether the discrepancy has been managed as expected. Successful management leads to positive affect, while persistent or worsening discrepancies result in negative affect (*Proposition 9*). Research on feedback processes supports this proposition (Fischer et al., 2021; Höpfner and Keith, 2021; Taylor, 1991).

### Contextual factors

It is essential to recognize that each stage is also influenced by various contextual factors, including, but not limited to, between-interaction factors such as self-regulation and knowledge (Gross, 2014; Baumeister and Vohs, 2004), between-person factors such as genetics (Lazarus, 1991), epigenetics, moral frameworks, personal experiences, socialization/culture, personality traits, and sociodemographic factors, as well as between-environment factors such as environmental demands (Bakker and Demerouti, 2007; Morgeson and Humphrey, 2006), resources, constraints, and other interactions the person is involved in. These contextual factors not only directly impact each stage (e.g., determine their expression) but also act as moderators between stages (e.g., determine whether stages proceed sequentially or in a compressed, fast-tracked manner). Therefore, our proposition is that each stage and its corresponding propositions are influenced by contextual factors—between-interaction, between-person, and between-environment—which can directly impact each stage and act as moderators between stages (*Proposition 10*). Research in intrapersonal regulation (Gross, 2015; Ochsner and Gross, 2005; Vohs and Heatherton, 2000), developmental and personality psychology (Deary et al., 2009; Hughes et al., 2020; Webster and Ward, 2011), and industrial-organizational psychology (Fisher et al., 2014; Grandey and Diamond, 2010; Oldham and Fried, 2016) support this proposition.

### Core proposition

In summary, the Interaction Discrepancy Model (IDM) posits that the dynamic and iterative process of person-environment interactions is defined by persons continuously striving to reconcile discrepancies between their current interactions (Is-State) and their expected interactions (Ought-State). This reconciliation occurs through a dynamic, iterative process involving the perception, appraisal, and response to these discrepancies.

## Discussion

### Critique of the model

#### Strengths of the interaction discrepancy model

The Interaction Discrepancy Model (IDM) presents several strengths, significantly advancing current theoretical models that explain person-environment interactions. Firstly, it adeptly predicts the dynamic, iterative, and feedback-driven nature of these interactions. By integrating the inherent characteristics of these interactions, the IDM aligns more closely with empirical evidence and lay observations.

The structured eight-stage approach of the IDM provides a detailed explanation of the latent mechanisms underlying person-environment interactions. This structure not only enhances our understanding but also allows for straightforward tests of falsifiability, strengthening its position as a robust explanatory framework.

Additionally, the IDM elucidates the complex interplay of cognitive, affective, and behavioral processes, explaining the varied cognitive, affective, and behavioral responses and adaptations observed in empirical research and daily life. This detailed approach offers a structured understanding of how these processes interact, capturing the wide variety of person-environment interactions.

Moreover, the IDM highlights the importance of non-conscious processes alongside conscious ones. This comprehensive view aligns with recent developments in neuroscience and cognitive psychology (e.g., Gazzaley and Nobre, 2012; Kozhevnikov et al., 2007; Nie et al., 2017), reinforcing both the academic and practical applicability of the model.

Finally, one of the IDM’s key strengths is its applicability across a wide range of contexts. It recognizes that person-environment interactions extend beyond interpersonal dynamics to include interactions with animals, inanimate objects, and abstract concepts. This broad applicability enhances the model’s relevance and utility in diverse fields.

#### Limitations of the interaction discrepancy model

Currently, the model may not apply to interactions among entities larger than individuals, such as groups or societies. Although the propositions in our model could potentially extend to these collective levels, a collective is different than the sum of its individual members (Baumeister et al., 2016). Consequently, factors like group dynamics may influence interactions at these levels, which are not captured by the current version of the IDM.

Furthermore, the IDM may encounter the same issue as other component process models (Scherer, 2009; Lazarus, 1991): the stages outlined in the IDM might occur in a different order. While prior research supports the assumption that these stages occur in the specified sequence (Scherer, 2000, 2009), the order may vary within and between person-environment interactions, between persons, and between different environments.

### Applications

The Interaction Discrepancy Model (IDM) has extensive applications due to its broad applicability to various person-environment interactions. Notably, the model can significantly contribute to research on aggression, delinquency, and crime. Traditional theories in these areas often attribute antisocial behaviors to stable constructs such as personality traits and sociodemographic factors (e.g., socioeconomic status, neighborhood environment, family violence, substance abuse; Meyer et al., 2024; Ullman et al., 2024; Weinberger, 2023). While we acknowledge the importance of these factors, our model emphasizes the substantial role of perceptions in triggering antisocial behaviors. Unlike existing models, the IDM suggests that such behaviors originate from a perceived discrepancy between an individual’s current interactions and their expected interactions. Consequently, aggression, delinquency, and crime are seen as responses to address these perceived discrepancies, aligning with research that indicates antisocial behaviors often have a moral justification (Burn and Brown, 2006; Harvey et al., 2017; Loza, 2007). Thus, it may be more effective to focus on modifying the perceptions and cognitive frameworks of individuals exhibiting antisocial behaviors rather than solely addressing their environmental contexts.

The IDM can also be applied to the study of conflict management, encompassing intra-group conflicts (e.g., group cohesion within organizational teams), inter-group conflicts (e.g., deadlock between political parties), intra-national conflicts (e.g., civil wars), and international conflicts (e.g., bilateral or proxy wars). Traditional models in this field often suggest that conflicts arise from competition over resources or deep-seated animosities (Burelli, 2021; Ross, 1986; Wendt, 1987). In contrast, our model proposes that hostilities primarily stem from perceived (moral) discrepancies, where at least one party holds another party responsible for an undesirable discrepancy. The aggrieved party attempts to rectify this discrepancy through belligerent actions. For instance, anger may drive retributive actions (e.g., suppression) when the discrepancy is perceived as changeable, such as in cases of separatism (Hagendoorn et al., 2008; Sharan, 2020; van Leeuwen and Mashuri, 2013). Conversely, disgust may drive (ex-)terminative actions (e.g., executions, genocides) when the discrepancy is perceived as unchangeable, such as differences in faith, nationality, or race (Bezo and Maggi, 2015; Seidman, 2013; Trevor-Roper, 2000).

Moreover, the IDM is applicable to personality psychology. The model suggests that individuals with certain personalities perceive person-environment interactions differently from those without such personalities. For example, individuals high in the dark tetrad traits (narcissism, Machiavellianism, psychopathy, and sadism) may perceive others’ actions as violating their moral frameworks while viewing their behaviors as morally justified. This perception divergence explains the antisocial behaviors commonly observed in these individuals (Book et al., 2015; Furnham et al., 2013; Harrison et al., 2018).

The IDM also has significant applications in psychopathology and psychotherapy. It implies that addressing only the symptomatology of psychopathologies, particularly mood disorders (American Psychiatric Association, 2022), is insufficient for reducing suffering. Instead, it is crucial to focus on and adapt patients’ perceptions and cognitive frameworks regarding their interactions with their environments. This approach also aligns with the principles of cognitive-behavioral therapies (Butler et al., 2006; Hofmann et al., 2012; Kazantzis et al., 2018).

Furthermore, the IDM can be effectively applied to the research areas of Industrial-Organizational psychology and organizational behavior. Our model provides compelling rationales for explaining why employees exhibit behaviors that align with organizational goals, such as organizational citizenship behavior (LePine et al., 2002; Ocampo et al., 2018; Podsakoff et al., 2000), which may result from a perceived sustainable discrepancy attributed to the organization and mediated by an autoregulatory response akin to the latent construct of love. Conversely, it also explains behaviors that conflict with organizational goals, such as counterproductive workplace behavior (Carpenter et al., 2020; Marcus et al., 2013; Wiernik and Ones, 2018), which may arise from a perceived changeable and undesirable discrepancy attributed to the organization and mediated by an autoregulatory response akin to the latent construct of anger. Additionally, the model elucidates why routine activities at work are sometimes performed with minimal cognitive and emotional responses and adaptations (see Eagle and Pentland, 2009), which may be due to a perceived congruence between current and expected interactions.

### Future research directions

#### Multi-stage validation

Our model presents several avenues for future research. Firstly, it is imperative to test the propositions outlined in this manuscript for internal validity. To potentially falsify the IDM (Popper, 1959), a multi-stage study could be conducted. Participants would be randomly assigned to different conditions based on the discrepancy between their current interaction and an expected interaction (desirable, undesirable, likely changeable, unlikely to change, uncertain outcomes, and a control group with no discrepancy). Following this assignment, each stage of the IDM could be tested consecutively. Participants could be exposed to various discrepancy conditions, and their perceptual and sensory responses could be monitored to test the proposition of Stage 1. It is crucial that the conditions are tailored to each participant, as reactions are subjectively relevant. The proposition of Stage 2 could be tested by evaluating the coherence and accuracy of the cognitive frameworks participants construct of the interactions. Stage 3 could be tested by asking participants about their perceived discrepancy condition and comparing it to their assigned condition. Stage 4 could be tested by observing whether participants in the discrepancy condition exhibit increased attentional focus on the discrepancy, while the control group continues routine behavior. Stage 5 could be tested by comparing the initially assumed appraisal components (valence, responsibility, changeability/sustainability) based on the initial discrepancy condition with the appraisal components the participant actually reported. Stage 6 could be tested by matching the appraisal outcomes with observed modulatory and motivational changes, measured through biomarkers and self-report surveys, and verifying whether the expected autoregulatory responses occur. Stage 7 could be tested by observing whether change-oriented emotions lead to change-oriented behaviors, mitigation-oriented emotions lead to mitigation-oriented behaviors, and sustainment-oriented emotions lead to sustainment-oriented behaviors. Stage 8 could be tested by manipulating the interaction with different types of feedback induction (overt, subliminal, control group with no feedback) and varying the success of the action plan (successful, unsuccessful, uncertain), then observing affective changes via self-report surveys and changes in Stages 1, 2, and 3 in the new interaction cycle. Finally, the influence of contextual factors could be examined by varying the degree to which participants are permitted to use self-regulation strategies and observing the impact on the mechanisms outlined in the IDM. Additionally, measuring participants’ predispositions and analyzing how these dispositions affect the IDM mechanisms would provide further insights. Exposing persons to different environments with varying levels of demands, resources, and constraints, and then assessing the impact on the IDM mechanisms, would also contribute to a comprehensive understanding of these contextual factors. It is advisable to initially test these propositions in an isolated and sequential manner. Any inconsistencies identified during these tests should be addressed, the model adjusted accordingly, and the entire 8-stage sequence subsequently tested. This approach would enable researchers to verify the internal validity of the Interaction Discrepancy Model (IDM) and its currently specified sequence of stages.

#### Collective-level validation

Finally, it is crucial to test the IDM’s propositions within collectives rather than individuals (Butler and Gross, 2009; Smith and Mackie, 2015; Thonhauser, 2022). This could involve examining how groups or societies interact with their environments and assessing whether these interactions significantly differ from those of individuals. In particular, interpersonal feedback mechanisms—as opposed to individual-level intrapersonal mechanisms—may be a key distinguishing variable. Accordingly, analyzing the role of group dynamics in these interactions may yield especially informative findings.

## Conclusion

Person-environment interactions have been the cornerstone of human experience, shaping our lives in profound ways. Despite their undeniable importance, the dynamic nature of these interactions has remained largely elusive. We aspire to change that. Through our proposed model, we aim to elucidate a rationale of how these interactions evolve, why they result in various cognitive, emotional, and behavioral responses, and how these processes influence each other. With every step forward, we move closer to deeper understanding of person-environment interactions.

## Data Availability

The original contributions presented in the study are included in the article/supplementary material, further inquiries can be directed to the corresponding author.
